# Solid-Nanoemulsion Preconcentrate for Oral Delivery of Paclitaxel: Formulation Design, Biodistribution, and *γ* Scintigraphy Imaging

**DOI:** 10.1155/2014/984756

**Published:** 2014-07-14

**Authors:** Javed Ahmad, Showkat R. Mir, Kanchan Kohli, Krishna Chuttani, Anil K. Mishra, A. K. Panda, Saima Amin

**Affiliations:** ^1^Department of Pharmaceutics, Faculty of Pharmacy, Jamia Hamdard, New Delhi 110062, India; ^2^Department of Phytochemistry, Faculty of Pharmacy, Jamia Hamdard, New Delhi 110062, India; ^3^Division of Cyclotron and Radiopharmaceutical Sciences, Institute of Nuclear Medicine and Allied Sciences (INMAS), New Delhi 110054, India; ^4^Product Development Cell-II, National Institute of Immunology (NII), New Delhi 110067, India

## Abstract

Aim of present study was to develop a solid nanoemulsion preconcentrate of paclitaxel (PAC) using oil [propylene glycol monocaprylate/glycerol monooleate, 4 : 1 w/w], surfactant [polyoxyethylene 20 sorbitan monooleate/polyoxyl 15 hydroxystearate, 1 : 1 w/w], and cosurfactant [diethylene glycol monoethyl ether/polyethylene glycol 300, 1 : 1 w/w] to form stable nanocarrier. The prepared formulation was characterized for droplet size, polydispersity index, and zeta potential. Transmission electron microscopy (TEM), differential scanning calorimetry (DSC), X-ray diffraction (XRD), and Fourier transform infrared spectroscopy (FTIR) were used to assess surface morphology and drug encapsulation and its integrity. Cumulative drug release of prepared formulation through dialysis bag and permeability coefficient through everted gut sac were found to be remarkably higher than the pure drug suspension and commercial intravenous product (Intaxel), respectively. Solid nanoemulsion preconcentrate of PAC exhibited strong inhibitory effect on proliferation of MCF-7 cells in MTT assay. *In vivo* systemic exposure of prepared formulation through oral administration was comparable to that of Intaxel in *γ* scintigraphy imaging. Our findings suggest that the prepared solid nanoemulsion preconcentrate can be used as an effective oral solid dosage form to improve dissolution and bioavailability of PAC.

## 1. Introduction

Oral administration of cancer therapeutics is attractive because of ease of administration to patients, but these molecules have poor oral bioavailability due to variable absorption and high drug effluxing through P-glycoprotein (P-gp) transporters in the lumen [1–3]. Paclitaxel (PAC) is one of the most potent chemotherapeutics. It is effective against wide spectrum of cancers such as ovarian cancer, breast cancer, head/neck cancer, and small and non-small cell lung cancers [4, 5]. Its recommended intravenous regimen is infusion over three hours every three weeks which is painful as well as causes hypersensitive reactions due Cremophor EL used as vehicle [5]. Its systemic bioavailability is less than 8% due to low aqueous solubility (0.3 ± 0.02 *μ*g/mL) [3, 6–8]. The low solubility is due to its highly lipophilic nature (log P 3.96) and bulky polycyclic structure (molecular weight 853 Da) [3]. The poor oral bioavailability is attributed to its significant first-pass metabolism by cytochrome P450 and P-gp mediated effluxing by intestinal cells [7]. Several formulation approaches including lipid-based nanocarriers [9, 10], prodrug [11], solid dispersions [12], polymeric nanoparticles [13], complexation with cyclodextrin [14], and pretreatment with P-gp inhibitors [15, 16] have been attempted to improve its* in vivo* and physicochemical properties but still its systemic exposure has not been increased.

In the present study, we attempted to develop robust and stable liquid-based nanocarrier for PAC using pharmaceutical excipients having P-gp modulation properties. These lipid-based nanoemulsion preconcentrates have attracted significant attention due to their promising ability to readily encapsulate the drug in nanocarrier immediately when it comes in contact with gastrointestinal fluid [17, 18]. Nanoemulsion preconcentrates are self-nanoemulsifying systems (SNES) which are composed of anhydrous isotropic mixtures of oil, surfactant(s), and cosurfactant(s) that spontaneously form oil-in-water nanoemulsions in GI tract by simple peristaltic movement [10, 18]. SNES can improve the bioavailability by circumventing the hepatic portal route, protecting drug against degradation in the harsh GI environment, facilitating intestinal lymphatic transport of drugs, decreasing cytochrome P450-induced metabolism, and inhibiting the P-gp mediated efflux due to intrinsic property of excipients used in formulation [19, 20]. Nevertheless, limited stability, low drug loading, and production issues often hinder its pharmaceutical application [21]. A solid-nanoemulsion preconcentrate is highly preferred due to its scalability and robustness, as well as its ability to avail all the benefits of a liquid system [21, 22]. Thus, this nanoparticulate system is anticipated to improve the bioavailability and therapeutic profile of PAC in the treatment of broad spectrum of cancers. Toward this goal, PAC loaded solid-nanoemulsion preconcentrate was formulated to improve the systemic exposure of PAC after oral administration. Propylene glycol esters and polyethylene glycol esters were used to prepare the liquid SNES. Polyoxyethylene (POE) was employed as an inert solid carrier to prepare solid-nanoemulsion preconcentrate of PAC by fusion method with the aim of investigating the potential of solid-nanoemulsion preconcentrate to improve the biodistribution of PAC through oral administration and its comparison with Intaxel, an intravenously given formulation of PAC.

## 2. Materials and Methods

### 2.1. Chemical and Reagents

PAC was a kind gift sample from Fresenius Kabi India Pvt. Ltd (Gurgaon, India). Intaxel (Commercial product of PAC in Cremophor EL/ethanol system) was purchased from Fresenius Kabi Oncology Ltd (Solan, India). Propylene glycol monocaprylate (Sefsol 218) was gifted by Nikko Chemicals (Tokyo, Japan), polyoxyl 15 hydroxystearate (Solutol HS 15) was obtained from BASF India Ltd (Mumbai, India), caprylocaproyl polyoxyl-8 glycerides (Labrasol), glyceryl monooleate (Peceol), glyceryl monolinoleate (Maisine 35-1), caprylic/capric triglycerides (Labrafac lipophile WL 1349), propylene glycol dicaprylocaprate (Labrafac PG), propylene glycol monolaurate (Lauroglycol FCC), polyglyceryl-6-dioleate (Plurol oleique), and diethylene glycol monoethyl ether (Transcutol HP) were provided by Gattefosse (Saint Priest Cedex, France). Polyoxyethylene (20) sorbitan monooleate (Tween 80), polyoxyethylene (20) sorbitan monolaurate (Tween 20), propylene glycol, polyethylene glycol 300 (PEG 300), and polyoxyethylene 4000 (POE 4000) were purchased from Merck (Mumbai, India). All other reagents are of analytical grades.

### 2.2. Formulation Design of Nanoemulsion Preconcentrate

The solubility of PAC in different components of nanoemulsion preconcentrate, oils, surfactants, and cosurfactants, was determined using shake flask method [23]. Samples were appropriately diluted with methanol and drug concentration was analysed by validated RP-HPLC method using acetonitrile-water (60 : 40) mobile phase with UV detection at 227 nm [24]. Further, pseudoternary phase diagrams were constructed to study the phase behavior of oil/surfactant/cosurfactant over the concentration ranges. Surfactant-cosurfactant mixture (Km) was dissolved in oil phase, in ratios 9 : 1 to 0.25 : 1 in glass vials at room temperature. The self-emulsification process of oil/surfactant/cosurfactant system was visually inspected after aqueous titration (spontaneous emulsification) method [25]. The percentage composition of each pseudoternary system was determined and observations were plotted on triangular coordinates to construct the phase diagrams. The self-emulsification region within this diagram was compared. On the basis of drug solubility and phase diagram studies, different components such as oil, surfactant, and cosurfactant were screened and nanoemulsion preconcentrate was prepared as reported previously [26]. Briefly, weighed quantity of drug (50 mg) was dissolved in different combinations of oil, surfactant, and cosurfactant (1 mL). The resulting mixture was vortexed to obtain a homogenous mass. The different combination of prepared nanoemulsion preconcentrate was sealed in transparent glass bottles and further evaluated for thermodynamic stability, dispersibility, percentage transmittance, and rate of self-emulsification [27].

### 2.3. Preparation of Solid-Nanoemulsion Preconcentrate

Solid-nanoemulsion preconcentrate was prepared by fusion method [28]. Accurately weighed amount of nanoemulsion preconcentrate and solid carrier (POE 4000) at the different weight-by-weight ratios of 1 : 1, 1.5 : 1, and 2 : 1 were mixed, respectively, at an elevated temperature of 60–70°C until a clear homogenous melt was obtained. The preparations were allowed to cool at room temperature to a consistent mass. The prepared solid-nanoemulsion preconcentrate was subsequently assessed for reconstitution characteristics.

### 2.4. Reconstitution Characteristics of Solid-Nanoemulsion Preconcentrate

#### 2.4.1. Visual Evaluation

The prepared solid-nanoemulsion preconcentrate, described above, was dispersed by mixing with 10 mL of distilled water for 30 s and then incubating for 10 min at 25°C. The performance of the formulations was visually assessed according to the grading system: a rapidly forming emulsion that is clear or slightly bluish in appearance was denoted by “A”; a rapidly forming, slightly less clear emulsion that has a bluish white appearance was denoted by “B”; bright white emulsion was denoted by “C”; a slightly oily, grayish white emulsion was denoted by “D” [26].

#### 2.4.2. Droplet Size, Polydispersity Index, and Zeta Potential Distribution

The average droplet size, polydispersity index (PDI), and zeta potential of the reconstituted nanoemulsions from the solid-nanoemulsion preconcentrate were assessed by Malvern Zetasizer (Nano ZS90, Malvern Instruments, UK). The sample was mixed with distilled water (1 : 100 v/v) before the analysis [26].

#### 2.4.3. Morphological Analysis

Morphology of the reconstituted nanoemulsions from the solid-nanoemulsion preconcentrate was observed by transmission electron microscopy (TEM; Morgagni 268D SEI, USA) operated at 200 kV and capable of point to point resolution. The preparation was diluted with water (1 : 100 v/v) before sample preparation. A drop of the reconstituted nanoemulsions was then directly deposited on the carbon coated grid and observed after drying [26].

### 2.5. Solid State Characterization of Solid-Nanoemulsion Preconcentrate

#### 2.5.1. Differential Scanning Calorimetry (DSC)

The physical state of PAC in the solid-nanoemulsion preconcentrate was characterized by DSC thermogram analysis (Pyris 6, PerkinElmer, USA). The samples included pure PAC, solid carrier (POE 4000), and the PAC loaded solid-nanoemulsion preconcentrate. The samples were placed in standard aluminum pans and then scanned at constant heating rate of 10°C/min between 20 and 450°C under a nitrogen gas flow of 20 mL/min [29].

#### 2.5.2. Fourier Transform Infrared Spectroscopy (FTIR) Analysis

The IR spectra were recorded on the FTIR (Jasco FT/IR-410, Japan) by using a potassium bromide pressed disc method. The spectrum was scanned over the frequency range of 4000–400 cm^−1^ [29].

#### 2.5.3. X-Ray Powder Diffraction (XRD)

To verify the physical state of PAC in solid-nanoemulsion preconcentrate, powder X-ray diffraction (XRD) patterns of the samples were recorded using X-ray diffractometer (Philips Xpert Pro), operated at 45 kV, 40 mA (CuK*α* radiation, *λ* = 1.5406 nm) with a scan speed of 0.02° s^−1^ in the 2*θ* range of 10°–80° [29].

### 2.6. *In Vitro* Drug Release Study

*In vitro* release test was performed as per USP XXIV method using 500 mL of freshly prepared dissolution medium, that is, simulated gastric fluid (SGF) pH 1.2 and simulated intestinal fluid (SIF) pH 7.4. The apparatus was set at 100 rpm and was maintained at 37 ± 0.5°C. The dissolution was carried out using dialysis bag (MWCO 12,000 g/mole; Sigma Aldrich, USA) [26]. The dialysis bag was pretreated by soaking it in the dissolution medium for 24 hours prior to commencement of each release experiment. PAC loaded solid-nanoemulsion preconcentrate was placed in dialysis bag containing 5 mL of the dissolution medium securely tied with a thermoresistant thread. 1 mL sample was withdrawn at regular time intervals (0.25, 0.5, 1, 2, 3, 4, 5, 6, and 8 h) and aliquot amount of dissolution medium was replaced to maintain sink condition. The release of drug from solid-nanoemulsion preconcentrate was compared to the drug suspension. The samples were analyzed for the drug content using HPLC method at 227 nm.

### 2.7. *Ex Vivo* Drug Absorption Study

*Ex vivo* intestinal transport study of optimized formulation was done by everted gut sac method. Animals were fasted overnight before the experiment. Everted sacs of albino Wistar rat ileum were prepared as described earlier [30]. The 10 cm intestinal segment was excised, tied at one end, everted with a smooth glass rod, and filled with 2 mL of Krebs-Ringer solution. The distended gut sac was placed in 50 mL of Krebs-Ringer solution containing 100 *μ*M PAC concentrations, continually aerated with 5% CO_2_ and 95% O_2_ and maintained at 37 ± 0.5°C. Aliquot of 100 *μ*L of serosal solution inside the sac was taken for quantitation of PAC permeated at different time intervals (0, 15, 30, 45, 60, 75, and 90 min) of the study. The study was done in triplicate and apparent permeability coefficient (*P*_app_) of solid-nanoemulsion preconcentrate was compared with commercial product Intaxel.

*P*_app_ was calculated with the following equation:

(1) $$\begin{array}{c} P_{\text{app}}=\frac{dQ}{dt}\times \frac{1}{AC_{0}}, \end{array}$$

where *dQ*/*dt* is the permeability rate, *C*_0_ is the initial concentration over the mucosal side, and *A* is the surface area.

### 2.8. Cell Cytotoxicity (MTT) Assay

MTT assay was used to assess the cytotoxicity of PAC alone, commercial product Intaxel, blank solid-nanoemulsion preconcentrate, and PAC loaded solid-nanoemulsion preconcentrate [31]. MCF-7 cells (ATCC, USA) were grown using DMEM (Dulbeccos modified eagle media) with 10% FBS (foetal bovine serum) and seeded on a single 96-well plate (Corning Costar, USA) and allowed to adhere to the wells [32]. The cells were then exposed to a series of concentrations of free PAC in less than 1% DMSO, commercial product Intaxel, blank solid-nanoemulsion preconcentrate, and PAC loaded solid-nanoemulsion preconcentrate. The final concentration of PAC was in the range of 0.0001–10 *μ*M. After 48 hours of treatment, the MTT assay was performed to check cell viability. The media were removed from all the wells and 10 *μ*L of MTT reagent (Chemicon International, USA) per well from a working stock (5 mg/mL) was added and incubated (37°C and 5% CO_2_) for 2-3 h. The reagent was then removed and the crystals were solubilised using isopropyl alcohol (IPA). It dissolves the formazan to give a homogeneous blue solution. The absorbance was measured at a wavelength of 570 nm (using 630 nm as reference wavelength) on ELISA plate reader (LMR-340 M, Labexim International, Austria). The value of absorbance is a measure of the number of live cells. The % cell viability was calculated with the following formula:

(2) $$\begin{array}{c} \%\;\;\text{cell}\;\;\text{viability}=\left(\frac{\text{Nt}}{\text{Nu}}\right)\times 100, \end{array}$$

where Nt is number of viable cells in treated group and Nu is number of viable cells in untreated group.

### 2.9. *In Vivo* Study

Animal study was performed according to a protocol submitted and approved by Institutional Animal Ethics Committee, Hamdard University (173/CPCSEA). The animals used for experiments were kept under standard laboratory conditions, at temperature 25 ± 2°C and relative humidity 55 ± 5%.

#### 2.9.1. Preparation of Radiolabeled Formulations

Radiolabeled ^99m^Tc-pertechnetate Intaxel solution and PAC loaded solid-nanoemulsion preconcentrate were prepared by adding 0.5 mL PAC formulation in sterile glass vial followed by addition of different concentrations of a reducing agent, stannous chloride (10–100 *μ*g), and pH was varied from 5 to 7 using 0.1 N NaHCO_3_. To the resultant mixture (filtered through 0.22 *μ*m membrane filter), required volume of ^99m^Tc-pertechnetate was added with continuous mixing and was incubated at 25 ± 5°C for 10 min and checked for radiolabeling efficiency [33].

#### 2.9.2. Determination of the Radiolabeling Efficiency, Radiochemical Impurity and Stability in Saline and Plasma

The radiolabeling efficiency was determined by thin layer chromatography method using the instant thin layer chromatography-silica gel (ITLC-SG) strips as stationary phase and acetone 100% as the mobile phase. After labeling of formulations, samples were placed on chromatographic paper. In this system, free pertechnetate migrates to the top of the plate, while formulation-attached material remains at the application point. The radiolabeling yield was expressed as a percentage of the total amount of radioactivity applied in the testing system [33]. Radiochemical impurity that is likely to exist in the form of unconjugated technetium (reduced free or hydrolyzed complex with water of the labeled entity) in ^99m^Tc-labeled PAC loaded solid-nanoemulsion preconcentrate and ^99m^Tc-labeled PAC-Intaxel solution was determined by ITLC-SG [34]. The effects of incubation time, pH, and stannous chloride concentration on labeling were studied to achieve optimum reaction conditions. The stability of radiolabeled formulation was evaluated in 0.9% (w/v) sodium chloride and in rat plasma. After the optimization and evaluation, stable radiolabeled-formulations of PAC were used for biodistribution study.

#### 2.9.3. Biodistribution Study

Wistar albino rats (female, aged 2-3 months) weighing between 250–280 g were selected for the study. Three rats for each formulation per time point were used in the study. Radiolabeled formulation, ^99m^Tc-PAC-solid nanoemulsion preconcentrate solution (equivalent to 10 mg/kg body weight), and commercial product ^99m^Tc-PAC-Intaxel solution (equivalent to 10 mg/kg body weight) were administered through oral route. Further, the radiolabeled complex of commercial product ^99m^Tc-PAC-Intaxel solution (equivalent to 2.5 mg/kg body weight) was injected through tail vein of Wistar rats. The rats were killed humanely at different time intervals and the blood was collected using cardiac puncture. Subsequently, heart, lungs, liver, spleen, kidney, stomach, and intestine were dissected, washed twice using normal saline, made free from adhering tissue/fluid, and weighed. Radioactivity present in each tissue/organ was measured using shielded well-type gamma scintillation counter (Caprac-R, Capintec, USA). Radio pharmaceutical uptake in each tissue/organ was calculated as a percentage of administered dose per gram of the tissue (% AD/g) using the below equation [33]:

(3) $$\begin{array}{c} \%\;\;\frac{\text{AD}}{\text{g}}=\frac{\text{Counts}\;\text{in}\;\text{sample}}{\text{Wt}\;\text{of}\;\text{sample}\times \text{Total}\;\text{counts}\;\text{administered}}\times 100. \end{array}$$

#### 2.9.4. Gamma Scintigraphy Imaging

Gamma (*γ*) scintigraphy imaging was performed on albino Wistar rats following intravenous administrations of radiolabeled commercial product Intaxel and oral administration of radiolabeled solid-nanoemulsion preconcentrate [33]. The study was carried out to evaluate the ability of developed formulation to gain systemic exposure of drug through oral route. Radiolabeled complex of developed formulation and ^99m^Tc-PAC-solid nanoemulsion preconcentrate solution (equivalent to 10.0 mg/kg body weight) was administered through oral route. Similarly, the radiolabeled complex of commercial product ^99m^Tc-PAC-Intaxel solution (equivalent to 2.5 mg/kg body weight) was injected through tail vein. The rats were anaesthetized using 0.5 mL ketamine (50 mg/mL) intramuscular injection and placed on the imaging board. Imaging was performed using single photon emission computerized tomography (SPECT, LC 75-005, Diacam, Siemens AG, Erlangen, Germany) gamma camera. The scintigraphy images following intravenous and oral administration of radiolabeled PAC containing formulation were recorded.

### 2.10. Statistical Analysis

The results are expressed as mean ± SD and were analyzed statistically (graph pad prism for Windows, version 5) using one-way analysis of variance (ANOVA) followed by Tukey's test and considered statistically significant when *P* < 0.05.

## 3. Results and Discussion

### 3.1. Preparation of Nanoemulsion Preconcentrate

The formulation components were selected based on the highest solubilizing capacity for PAC and ability of the preliminary pseudoternary system to form stable nanoemulsion at minimum concentration of surfactant for the maximum lipid content. Phase behavior of the pseudoternary system was used to optimize the different formulation components such as oil phase [propylene glycol monocaprylate/glycerol monooleate (4 : 1 w/w)], surfactant [polyoxyethylene 20 sorbitan monooleate/olyoxyl 15 hydroxystearate (1 : 1 w/w)], and cosurfactant [diethylene glycol monoethyl ether/polyethylene glycol 300 (1 : 1 w/w)] to form stable nanocarrier for PAC. Nanoemulsion preconcentrate with different concentration of these optimized components was evaluated for thermodynamic stability, dispersibility, percentage transmittance, and rate of self-emulsification (Table 1). Among the various combinations, composition S_4_ having 25% of oil phase, 50% of surfactants, and 25% of cosurfactants was observed to have good thermodynamic stability, emulsification rate, and high percent transmittance. Upon optimization, it was found to have minimum droplet size (41.10 nm) and 82.85% cumulative drug release after 4 h [35].

### 3.2. Preparation of Solid-Nanoemulsion Preconcentrate

**Figure 1:**
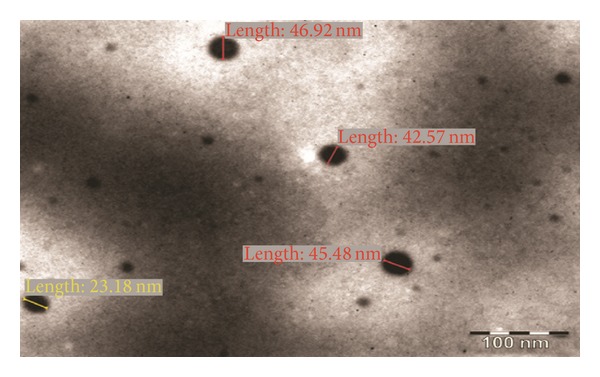
TEM image of PAC loaded solid-nanoemulsion preconcentrate.

**Figure 2:**
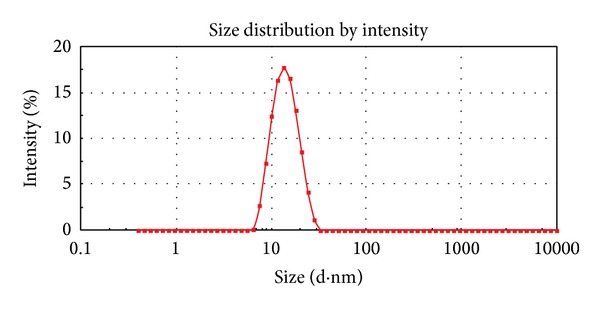
Droplet size distribution of PAC loaded solid-nanoemulsion preconcentrate.

**Figure 3:**
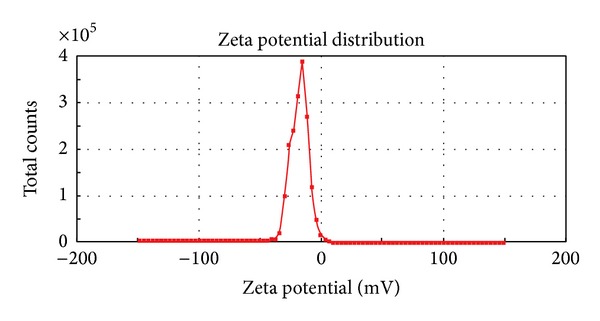
Zeta potential distribution of PAC loaded solid-nanoemulsion preconcentrate.

The nanoemulsion preconcentrate (formulation S_4_) was selected to prepare PAC loaded solid-nanoemulsion preconcentrate with the aid of POE 4000 as a carrier. It was found that when the isotropic mixture of formulation S_4_ was dispersed in molten POE 4000 by fusion method and then cooled to room temperature, a consistent solid matrix was formed, which was reconstituted to a nanoemulsion upon dilution with aqueous phase. The solid consistency of formulation was formed at 40% (w/w) or higher concentration of POE 4000. Solid-nanoemulsion preconcentrate composed of formulation S_4_ and POE 4000 in ratio of 1.5 : 1 was found optimum to be designed into solid carrier after self-emulsification performance, solid state consistency, and droplet size analysis with mean droplet size of 47.33 nm and minimum PDI (0.125). Furthermore, this optimized composition of solid-nanoemulsion preconcentrate was found to preserve the self-emulsifying property of formulation S_4_ and the dispersibility in “Grade A” category. The globule morphology of nanoemulsion upon reconstitution was observed to be spherical (Figure 1) and globule size was in good agreement with droplet size distribution analyzed by photon correlation spectroscopy (Figure 2). The zeta potential was −23.2 mV, which indicated the desirable stability and negatively charged surface of drug carrier (Figure 3). The presence of high negative charge could be due to presence of anionic group of fatty acids and glycols present in the oil, surfactant, cosurfactant, and solid carrier [36].

### 3.3. Characterization of Solid-Nanoemulsion Preconcentrate

#### 3.3.1. Differential Scanning Calorimetry (DSC)

**Figure 4:**
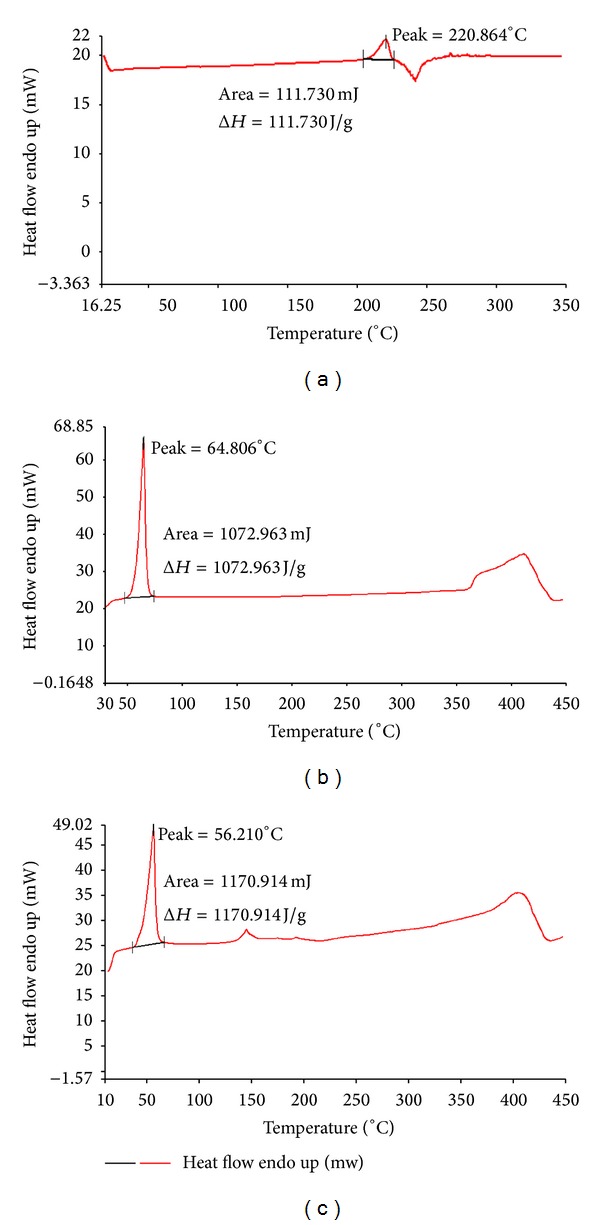
DSC thermogram of (a) paclitaxel, (b) POE 4000, and (c) PAC loaded solid-nanoemulsion preconcentrate.

The DSC technique was used to acquire qualitative information about the physicochemical status of drug in the solid-nanoemulsion preconcentrate. The thermograms of PAC, POE 4000, and PAC loaded solid-nanoemulsion preconcentrate are shown in Figure 4. PAC powder showed a sharp endothermic peak at 220.8°C that was correlated to the melting point of the drug in the crystalline form. No distinct peaks were observed for POE 4000 near this range of temperature. However, POE 4000 showed an endothermic peak at 64.8°C corresponding to its melting point. The thermogram of PAC loaded solid-nanoemulsion preconcentrate did not show the characteristic endothermic peak of drug, suggesting that the drug is either completely incorporated or molecularly dispersed in the amorphous state in the solid matrix [37].

#### 3.3.2. Fourier Transform Infrared Spectroscopy (FTIR) Analysis

**Figure 5:**
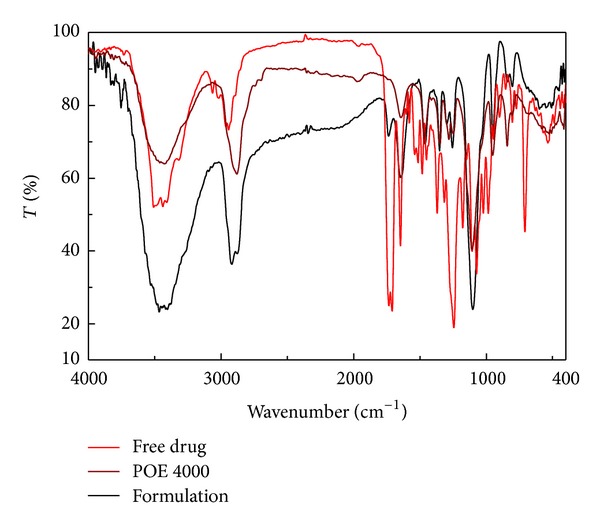
FTIR spectra of paclitaxel (in red), POE 4000 (in brown), and PAC loaded solid-nanoemulsion preconcentrate (in black).

The IR spectrum of PAC, POE 4000, and PAC containing solid-nanoemulsion preconcentrate was recorded and the characteristic peaks were compared for drug-excipient interaction study (Figure 5). The PAC alone showed two carbonyl absorption bands at 1733 cm^−1^ and 1646 cm^−1^, assigned to carbonyl-carbonyl and amide-carbonyl stretching, respectively. These bands are of diagnostic value to elucidate drug interaction with excipient. The carbonyl band of acid (1736 cm^−1^) and amide (1647 cm^−1^) stretching of PAC in solid-nanoemulsion preconcentrate indicated that there was no interaction between drug and formulation excipients. These illustrations of IR spectra of PAC alone and PAC in solid-nanoemulsion preconcentrate suggest absence of chemical drug-carrier interaction [29].

#### 3.3.3. X-Ray Powder Diffraction (XRD)

**Figure 6:**
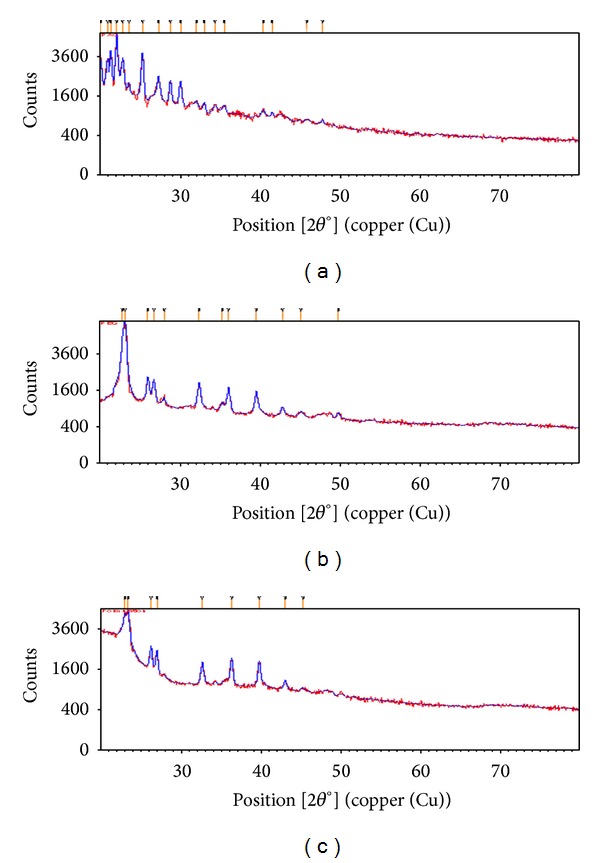
X-ray diffraction pattern of (a) paclitaxel, (b) POE 4000, and (c) PAC loaded solid-nanoemulsion preconcentrate.

The molecular dispersion of drug in the solid-nanoemulsion preconcentrate was further verified by powder-XRD analysis. Typical diffraction patterns of PAC, POE 4000, and PAC containing solid-nanoemulsion preconcentrate are shown in Figure 6. The diffraction patterns of pure PAC revealed a highly crystalline structure. These typical patterns were absent in the PAC containing solid-nanoemulsion preconcentrate, indicating that the encapsulated drug is either molecularly dispersed or is in the amorphous state in the matrix [38].

DSC and XRD analysis for the physical mixture of PAC and PEO 4000 with a similar ratio as in formulation S_4_ were carried out. DSC thermogram showed small endothermic peak for PAC at 220°C and high endothermic peak for PEO 4000 at 63°C. The XRD analysis of physical mixture with similar ratio as in the formulation showed closed pack, multiple peaks characteristic of PAC with 2*θ* between 10 and 30° as well as of PEO 4000 with 2*θ* of 23.30°, 26.17°, and 28.8° along with additional 2*θ* at 32.50, 36.18, and 39.61, which was absent in case of PAC alone. This suggests that the drug was adequate with respect to PEO 4000 in the mixture but was completely encapsulated by the polymer.

### 3.4. *In Vitro* Drug Release Study

**Figure 7:**
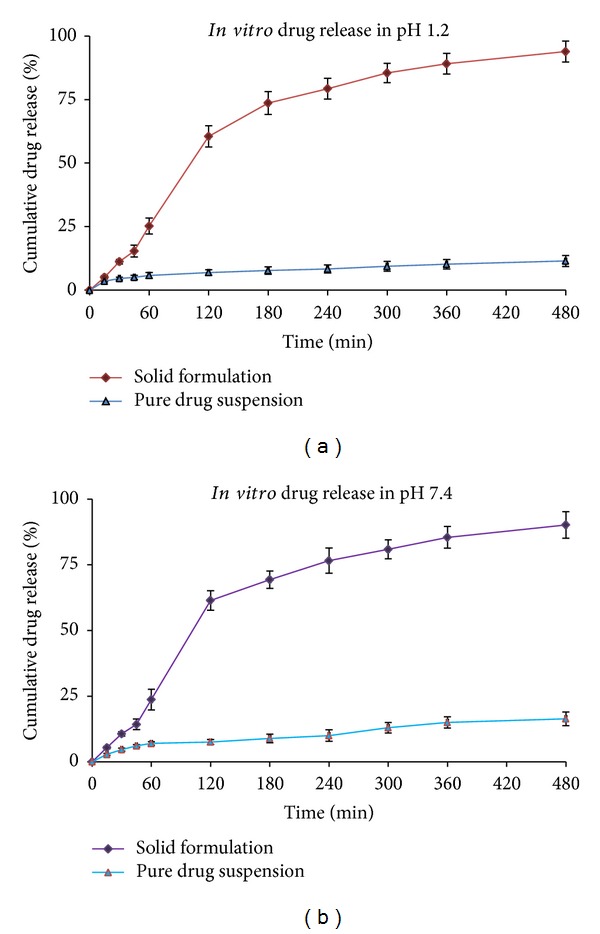
(a)* In vitro* drug release profile in dissolution media pH 1.2. (b)* In vitro* drug release profile in dissolution media pH 7.4.

Conventional* in vitro* dissolution studies are not preferred for nanoemulsion preconcentrate because when such a three-component system comes in contact with water, then drug exists as free molecules, as micelles, or as nanoemulsion droplets. Under such conditions, it is desirable to separate the isolated drug molecules from the trapped drug molecules as micelles or as nanoemulsion. The use of dialysis bag technique provides consistent drug dissolution for such formulations [39]. The percentage dissolution time profile of the solid-nanoemulsion preconcentrate in SGF (pH 1.2) as well as SIF (pH 7.4) is shown in Figures 7(a) and 7(b). It was observed that the release of drug from solid-nanoemulsion preconcentrate in both of the dissolution media remains almost similar. After 4 h, the percentage cumulative drug release from the solid-nanoemulsion preconcentrate was almost 75%. The percentage dissolution profile of solid-nanoemulsion preconcentrate was compared with pure drug suspension (aqueous suspension of PAC) and was found to be higher. It is observed that solid-nanoemulsion preconcentrate will remain as nanosized droplet in the gastric fluid and consequently would show systemic absorption by lymphatic drainage route in gastric region. However, a small fraction of drug may leach out of the emulsified system but would be absorbed as drug micelles due to presence of surfactant-cosurfactant. It is also probable that if the concentration of surfactant is insufficient to form micelles, then PAC would not be absorbed at all (as in case of aqueous drug suspension) because it is a BCS class IV drug.

### 3.5. *Ex Vivo* Drug Absorption Study

Everted gut sac study was performed with PAC containing solid-nanoemulsion preconcentrate and the commercial product Intaxel. *P*_app_ of the PAC loaded solid-nanoemulsion preconcentrate (6.71 × 10^−5^ cm/s) was higher than that of Intaxel (1.18 × 10^−5^ cm/s). This enhancement could be attributed to the P-gp inhibitory effect of the formulation excipients (Peceol, Solutol HS-15, Tween 80, and polyethylene glycol or polyoxyethylene) in addition to encapsulation of drug inside the nanocarrier.

### 3.6. Cell Cytotoxicity Assay

**Figure 8:**
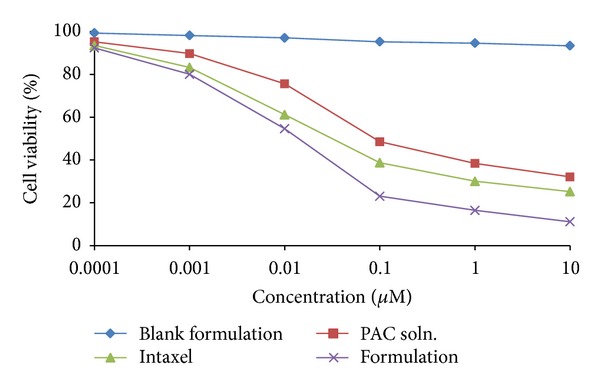
Inhibitory effect of various paclitaxel treatments on the proliferation of MCF-7 cells.

*In vitro* cytotoxicity using MCF-7 was evaluated for PAC alone, commercial product Intaxel, solid-nanoemulsion preconcentrate, and PAC loaded solid-nanoemulsion preconcentrate in the concentration range of 0.0001–10 *μ*M.  Figure 8 represents the inhibitory effect of PAC on the proliferation of MCF-7 cells. At various concentration points, PAC loaded solid-nanoemulsion preconcentrate exhibited the strong inhibitory effect on the proliferation of MCF-7 cells. This suggests that incorporation of PAC in solid-nanoemulsion preconcentrate potentiates the cytotoxic propensity. The percentage cell viability at 0.1 *μ*M (23.08%) was found to be significantly low (*P* < 0.001) compared to other treatments. It was due to the increased intracellular uptake of PAC in MCF-7 cells.

### 3.7. *In Vivo* Study

#### 3.7.1. Radiolabeling Experiments

Radiolabeling of the commercial product Intaxel and PAC-solid nanoemulsion preconcentrate was optimized at variable pH range of 5–7 and highest labeling efficiency was observed at pH 6.5. The amount of stannous chloride (reducing agent) plays a very decisive role in determining extent of labeling. At 50 *μ*g of stannous chloride, highest labeling efficiency and minimum radiocolloids were obtained. The stability of radiolabeled Intaxel as well as PAC-solid nanoemulsion preconcentrate was evaluated in normal saline and plasma (Table 2). Results of stability studies showed the radiolabelled products to be stable over 24 h of period and suitable for biodistribution study as well as scintigraphy imaging.

#### 3.7.2. Biodistribution Study

**Figure 9:**
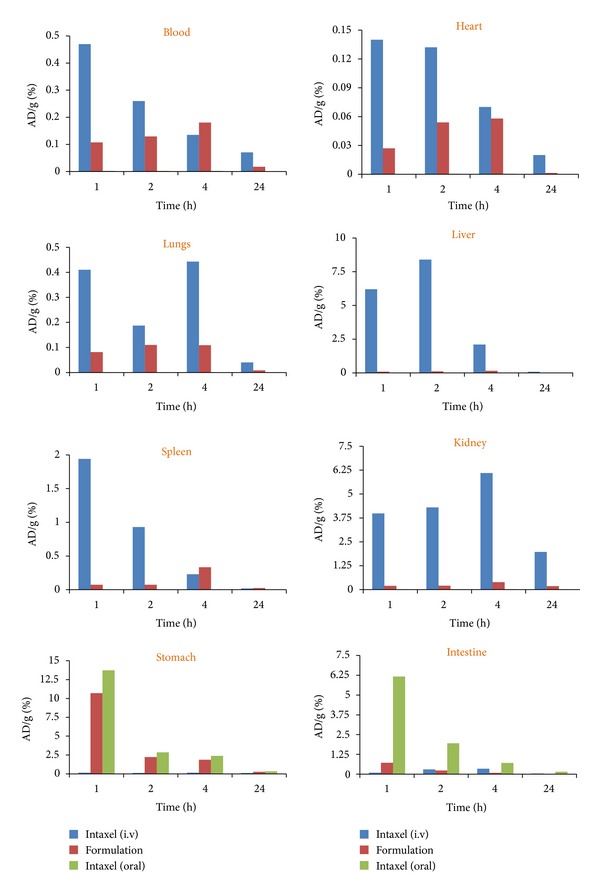
Biodistribution pattern of radiolabeled PAC formulation (Intaxel and solid-nanoemulsion preconcentrate) in various tissues at different time intervals.

The tissue distribution profile of radiolabeled PAC-solid nanoemulsion preconcentrate was compared to radiolabeled commercial product Intaxel in various tissues such as blood, heart, lungs, liver, spleen, kidney, stomach, and intestine (Figure 9). The radio counts in different tissues were estimated at different time intervals of 1, 2, 4, and 24 h after intravenous and oral administration of radiolabeled formulation. Figure 9 illustrates that encapsulation of PAC into solid-nanoemulsion preconcentrate helps to gain systemic exposure of drug through oral route. However, oral administration of commercial intravenous product Intaxel showed significantly low (*P* < 0.001) systemic exposure in comparison to solid-nanoemulsion preconcentrate. The enhancement in systemic exposure of PAC loaded solid-nanoemulsion preconcentrate is due to increase in permeability of drug as lipidic nanocarrier as well as inhibition of P-gp mediated effluxing by formulation excipients.

#### 3.7.3. *γ* Scintigraphy Imaging

**Figure 10:**
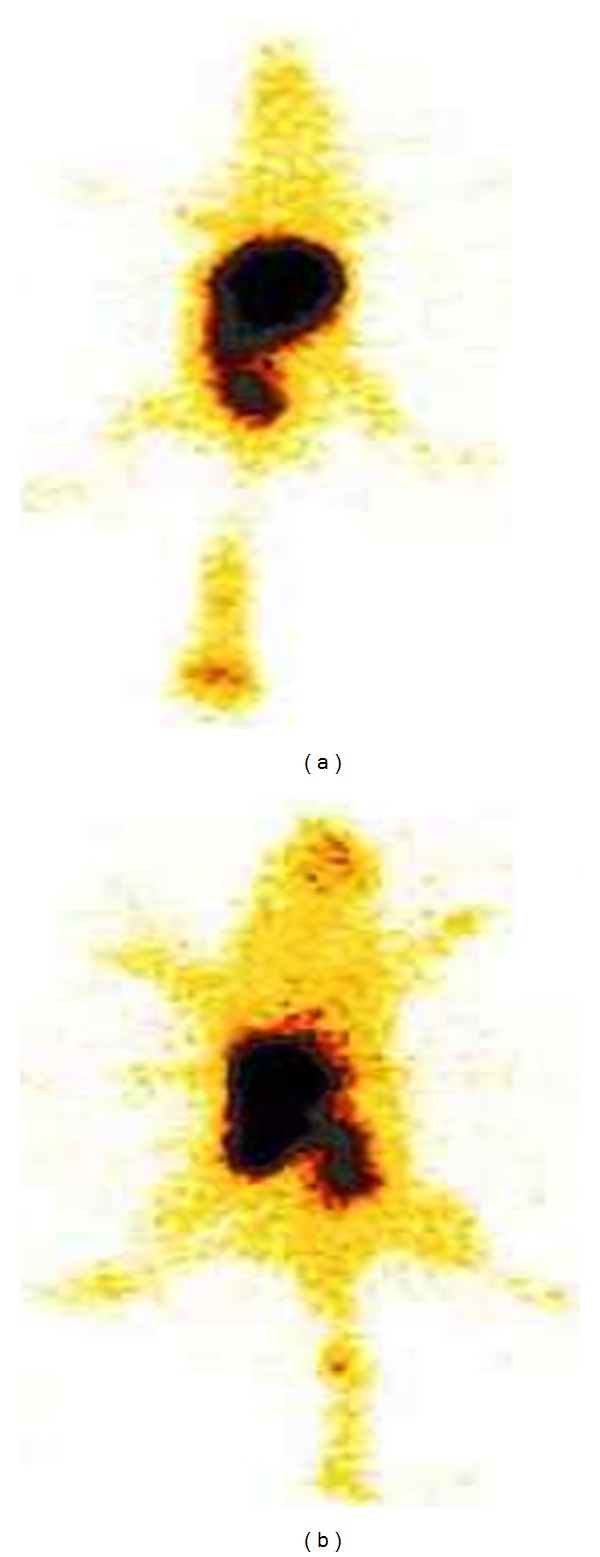
Pharmacoscintigraphic evaluation of radiolabeled PAC formulation after 2 h: (a) i.v. administration of ^99m^Tc labeled Intaxel and (b) oral administration of ^99m^Tc labeled solid-nanoemulsion preconcentrate.

The gamma scintigraphy images in rat at 2 h after intravenous and oral administration of radiolabelled formulation are shown in Figure 10. Upon oral administration of radiolabeled solid-nanoemulsion preconcentrate, the systemic exposure of radioactivity was equivalent to the intravenous administration of commercial product Intaxel indicating the high absorption of PAC loaded solid-nanoemulsion preconcentrate after oral administration.

## 4. Conclusion

Solid-nanoemulsion preconcentrate system seems to be a good approach to increase the bioavailability for hydrophobic drugs such as paclitaxel. In the present study, the solid-nanoemulsion preconcentrate of paclitaxel has shown good thermodynamic stability with prompt emulsification and optimum drug release in the biological fluid. The spherical globule size obtained in nanometric range is responsible for its high permeation across the biological membrane. Such systems will offer greater stability and efficacy for anticancer drugs.

## Figures and Tables

**Table 1:** Observation of different composition of nanoemulsion preconcentrate for thermodynamic stability, dispersibility, percentage transmittance, and rate of emulsification.

Formulation	Composition [oil : surfactant : cosurfactant] (*µ*L)	Centrifugation cycle	Heating-cooling cycle	Freeze-thaw cycle	Dispersibility test		Percentage transmittance	Emulsification rate (sec)
					0.1 N HCl	Distilled water		
S1	175 : 300 : 225	*√*	*√*	*√*	Grade A	Grade A	92.05	30
S2	225 : 250 : 175	*√*	x	x	Grade B	Grade B	76.23	45
S3	200 : 350 : 125	*√*	*√*	*√*	Grade A	Grade A	89.43	52
S4∗	175 : 350 : 175	*√*	*√*	*√*	Grade A	Grade A	99.55	38
S5	200 : 300 : 175	*√*	*√*	*√*	Grade B	Grade B	85.34	42
S6	175 : 300 : 125	*√*	*√*	*√*	Grade A	Grade A	87.91	47
S7	225 : 300 : 125	*√*	*√*	*√*	Grade B	Grade B	82.67	50
S8	175 : 250 : 175	*√*	*√*	*√*	Grade B	Grade B	84.18	35
S9	225 : 350 : 175	*√*	*√*	*√*	Grade B	Grade B	87.31	46
S10	200 : 250 : 125	*√*	*√*	*√*	Grade B	Grade B	75.84	44
S11	200 : 250 : 225	*√*	*√*	*√*	Grade B	Grade B	76.09	40
S12	225 : 300 : 225	*√*	*√*	*√*	Grade B	Grade B	81.49	48
S13	200 : 350 : 225	*√*	*√*	*√*	Grade A	Grade A	97.06	40

*Indicates optimized formulation.

**Table 2:** Stability of radiolabeled complexes of PAC formulations (^99m^Tc-Intaxel and ^99m^Tc-PAC loaded solid-nanoemulsion preconcentrate) in saline and plasma at different time intervals.

Time (h)	Radiolabeling efficiency (%)			
	Intaxel		PAC loaded solid- nanoemulsion preconcentrate	
	Saline	Plasma	Saline	Plasma
1	95.63 ± 0.24	95.14 ± 0.37	96.85 ± 0.59	96.48 ± 0.26
2	94.72 ± 0.51	94.22 ± 0.41	95.69 ± 0.43	94.91 ± 0.39
4	94.09 ± 0.33	93.56 ± 0.60	95.05 ± 0.72	94.54 ± 1.07
8	93.45 ± 0.86	92.67 ± 1.16	94.17 ± 1.14	93.76 ± 0.96
24	91.86 ± 1.05	90.08 ± 0.93	92.94 ± 0.88	92.11 ± 1.28
